# Aloe Emodin Reduces Cardiac Inflammation Induced by a High-Fat Diet through the TLR4 Signaling Pathway

**DOI:** 10.1155/2020/6318520

**Published:** 2020-02-05

**Authors:** Yingfu Chen, Burong Feng, Ye Yuan, Juan Hu, Wei Zhao, Huiwei Jiang, Wen Li, Ziyi Fan, Zhimin Du

**Affiliations:** ^1^Institute of Clinical Pharmacy, The Second Affiliated Hospital of Harbin Medical University (The University Key Laboratory of Drug Research, Heilongjiang Province, Harbin 150086, China; ^2^Department of Clinical Pharmacology, College of Pharmacy, Harbin Medical University, Harbin 150086, China; ^3^State Key Laboratory of Quality Research in Chinese Medicines, Macau University of Science and Technology, Macau, China

## Abstract

**Background:**

Aloe emodin (AE) is a lipid-lowering agent, which could be used to treat hyperlipidemia, thereby reducing the risk of cardiovascular disease. Recent evidence suggests that hyperlipidemia is associated with many cardiac pathological alterations and might worsen myocardial damages.

**Purpose:**

The purpose of this study is to evaluate the potential roles and mechanisms of AE in hyperlipidemia-induced oxidative stress and inflammation in the heart. *Study Design*. We established a hyperlipidemia-induced cardiac inflammation model in rats and cells then administered AE and observed its effect on hyperlipidemia-induced cardiac inflammation.

**Methods:**

We used a mouse model of hyperlipidemia caused by a high-fat diet (HFD) for 10 weeks and cell culture experimental models of inflammation in the heart stimulated by PA for 14 h. Inflammatory markers were detected by qRT-PCR, WB, and immunofluorescence.

**Results:**

We demonstrated that the expression levels of proinflammatory cytokines IL-1*β*, IL-6, and TNF-*α* were increased in the HFD group compared to the normal diet (ND) group, whereas AE treatment significantly reduced their levels in the myocardium. In addition, vascular cell adhesion molecule 1 (VCAM1) and intercellular adhesion molecule 1 (ICAM-1) protein expressions were also inhibited by AE. Our *in vitro* study showed AE treatment dose-dependently decreased the expression of IL-1*β*, IL-6, and TNF-*α* were increased in the HFD group compared to the normal diet (ND) group, whereas AE treatment significantly reduced their levels in the myocardium. In addition, vascular cell adhesion molecule 1 (VCAM1) and intercellular adhesion molecule 1 (ICAM-1) protein expressions were also inhibited by AE. Our *κ*B, and p-P65l *in vivo* and *in vitro* study showed AE treatment dose-dependently decreased the expression of IL-1

**Conclusion:**

Taken together, our findings disclose that AE could alleviate HFD/PA-induced cardiac inflammation via inhibition of the TLR4/NF-*κ*B signaling pathway. Thus, AE may be a promising therapeutic strategy for preventing hyperlipidemia-induced myocardial injury.*κ*B, and p-P65l

## 1. Introduction

With the rapid development of our national economy, high-fat diet (HFD) is becoming increasingly common in many countries; epidemiological studies have suggested that continuous HFD will lead to hyperlipidemia [1, 2]. Hyperlipidemia is mainly characterized by decreased HDL levels and elevated levels of total cholesterol (TC), triglyceride (TG), and LDL. Clinical trials and observational studies have identified hyperlipidemia plays a key role in the development of cardiovascular diseases (CVD) [3, 4].

Results of recent studies suggest that the development of chronic inflammation is associated with hyperlipidemia [5, 6]. Signs of inflammation occur hand-in-hand with incipient lipid accumulation in the artery wall [7]; excessive lipoprotein levels trigger inflammation by modulating leukocyte activity and disturbing cytokine regulation [8]. Moreover, it is reported that the development of chronic inflammation will lead to type II diabetes mellitus, NAFLD (nonalcoholic fatty liver disease), hepatic fibrosis, and cardiovascular diseases [9]. Nowadays, chronic cardiac inflammation has become a major threat to the health of people. Excess saturated fatty acids cause deleterious changes in the lipid composition of plasma membranes, which induce the generation of reactive oxygen species (ROS). In addition, hyperlipidemia could elicit the innate and adaptive immune responses, accompanied by an expansion of myeloid cells and aggravation of ROS release inflammation, accelerating the progression of atherosclerosis [10].

Toll-like receptor 4 (TLR4) is an important modulator of innate immunity which is associated with innate immunity and metabolic disorders including hyperlipidemia [11]; TLR4 is essential for inflammation development [12]. So far, how HFD and other metabolic factors activate TLR4-dependent innate immune response remains an open question, but HFD seems to trigger acute and chronic inflammation through the TLR4 signaling pathway [13, 14]. Therefore, effectively mediated hyperlipidemia and proinflammatory cytokines have become a crucial issue in HFD-induced cardiac inflammation.

Plants have been utilized by humans from the very beginning of human existence. People have familiarized themselves with plants and used them in a variety of ways throughout the ages. In the 21st century, the pharmacological effects of medicinal plants have been regarded as a promising future drug/medicine for the management of health care [15]. As a member of the anthraquinone family, AE occurs in the leaves of aloe and exhibits a variety of pharmacological properties, such as antibacterial, anticancer, and antioxidant activities. Recently, AE has been reported to have anti-inflammatory effects [16]; in addition, AE previously showed a lipid-lowering effect in vivo [17]. However, the effect of AE on hyperlipidemia-induced cardiac inflammation has not been reported. Based on that fact, the aim of the present study is to investigate the potential roles and mechanisms of AE in hyperlipidemia-induced oxidative stress and inflammation.

## 2. Material and Methods

### 2.1. Animal Model

Healthy adult male Wistar rats (180-200 g) were obtained from the Animal Center of the Second Affiliated Hospital of Harbin Medical University (Harbin, China), used in the present, and were kept under standard animal room conditions (temperature 23 ± 1°C; humidity 55 ± 5%) with food and water ad libitum for 2 weeks before the experiments. The study was approved by the Animal Care and Use Committee of Harbin Medical University. All experimental procedures were performed in accordance with the Guide for the Care and Use of Laboratory Animals, published by the US National Institutes of Health (NIH Publication, 8th Edition, 2011).

First, rats were randomly divided into the normal diet group and high-fat diet group. HFD consists of maintaining feed 77.6%, lard 10%, yolk powder 10%, cholesterol 2%, bile salt 0.2%, and methylthiouracil 0.2%, purchased from HuaFuKang Biotechnology Co., Ltd. (Beijing, China). After 4 weeks, the serum lipid indexes were determined to confirm if the high-fat model has been established or not. The experimental model of HFD was divided into four groups: HFD group, AE (50 mg/kg) group, AE (100 mg/kg) group, and ATO group (7.2 mg/kg). After the establishment of the hyperlipidemia model, the animals were sacrificed, and blood samples were collected for serum preparation after 6 weeks of administration. The blood samples were used for the measurement of TC (total cholesterol), LDL (low-density lipoprotein), HDL (high-density lipoprotein), and TG (triglyceride) levels. The heart tissues were weighed and fixed with 4% paraformaldehyde for histological analysis or frozen in liquid nitrogen for further experiments including protein and gene level determinations.

### 2.2. Reagents

Palmitic acid was purchased from Sigma-Aldrich (St. Louis, MO); stock solutions of 5 mM PA/10% BSA were prepared and stored at -20°C. The stock complex solution was heated for 10 min at 55°C and then cooled to room temperature prior to use. AE, obtained from Xi'an Natural Field Bio-technique Co., Ltd., was dissolved in dimethyl sulfoxide (DMSO). Kits of TC, LDL, HDL, and TG were purchased from Nanjing Jiancheng Bioengineering Institute. The antibodies used in this study with their working dilutions indicated in parentheses: ICAM-1 (1 : 500), NF-*κ*B P65 subunit (1 : 500), TLR4 (1 : 500), and I*κ*B (1 : 500), were purchased from Proteintech Group, Inc. (Wuhan, China); p-NF-*κ*B P65 (1 : 1000) was purchased from Cell Signaling Technology (Danvers, MA) and VCAM-1 (1 : 500) from Bioss Antibodies (Beijing, China). The secondary antibodies were all from LI-COR Biosciences Co., Ltd.

### 2.3. Cell Culture

The H9C2 embryonic rat heart-derived cell line was obtained from the Department of Pharmacology of the Second Affiliated Hospital of Harbin Medical University, cultured with Dulbecco's modified Eagle medium (DMEM, Corning, USA) and 10% fetal bovine serum (ExCell Bio, China). The cardiomyocytes were cultured at 37°C with 5% CO_2_ after their isolations.

### 2.4. Molecule Docking

Preprocessing of ligand molecules involved the conversion of the dimension from 2D to 3D and conversion of the file format to pdb using ChemOffice. In order to keep the receptor and ligand in the same coordinate space, Python and AutoDockTools-1.5.6 are used to convert the receptor and ligand; the center of the active site is used for the conversion of receptor coordinates, and the geometric center of the ligand is used to transform the molecule to the same coordinate space. The size of the grid box in AutoDock Vina was kept as 40, 40, and 40 for X, Y, and Z; the binding affinity of ligands was negative in Kcal/mol. The AutoDock Vina script generates nine ligand postures for each ligand input, and each ligand has different binding energies. Extraction of ligands with optimal binding affinity from docking complexes using internal Perl scripts and the 3D structure of proteins and ligands are generated by PyMol.

### 2.5. Assay of Oxidative Stress

The intracellular reactive oxygen species (ROS) level was detected with 2,7-dichlorodihydro fluorescent diacetate (DCFH-DA). First, cells were pretreated with different concentrations of AE (25 *μ*M, 50 *μ*M, and 100 *μ*M) for 1 h, then incubated with PA (500 *μ*M) for 14 h. Finally, cells were loaded with 2 *μ*M DCFH-DA, incubated at 37°C for 30 min, and washed three times with Phosphate-Buffered Saline Tween-20 (PBST). The fluorescence intensity was determined with a fluorescence microscope, with excitation wavelengths of 488 nm. A commercial ELISA kit (Nanjing Jiancheng Bioengineering Institute) was used to analyze Superoxide Dismutase (SOD) according to the manufacturer's instructions.

### 2.6. Immunofluorescence Staining

The cells were lined in the culture plate with glass slides; the cells were fixed with 4% paraformaldehyde (PFA) at room temperature for 15 min. The PFA was discarded and washed with Phosphate-Buffered Saline (PBS) 3 times for 10 min each. The permeabilized cells with 0.3% Triton X-100 were washed and incubated with 5% bovine serum albumin (BSA) for 1 h; the cells were then incubated with the primary antibodies for intercellular cell adhesion molecule 1 (ICAM-1) and vascular cell adhesion molecule 1 (VCAM-1) subunit (1 : 200) overnight at 4°C. The cells were then washed and incubated in the dark at room temperature for 1 h with secondary antibodies including Alexa Fluor 488-conjugated Goat anti-Rabbit IgG (H+L) (Life Technologies) and finally incubated with 4′,6-diamidino-2-phenylindole (DAPI, Beyotime, Haimen, China) for 5 min at room temperature in the dark to counterstain the nuclei. Images were obtained using an Olympus microscope (Japan).

### 2.7. Live-Dead Cell Staining

The LIVE/DEAD ® Viability/Cytotoxicity Assay Kit (Invitrogen, Carlsbad, CA, USA) was used to analyze the amount of live and dead cells. H9C2 cells were cultured and treated with AE and PA. The mixture of dye calcein acetoxymethyl ester (calcein-AM, 0.5 *μ*l/ml) with ethidium homodimer-1 (EthD-1, 2 *μ*l/ml) was added to 6-well plates followed by incubation for 15 min. The quantity of live and dead cells was determined with a Laser Scanning Confocal Microscope (FV1000, Olympus, Japan). The ratio of dead cells to total cells was calculated for quantitative comparisons.

### 2.8. Histological Analyses

The heart tissue mass was fixed with 10% formaldehyde for 24 h and then gradient dehydration with sucrose. The samples were coated with OCT encapsulating adhesive after dehydration and frozen in a refrigerator at -80°C overnight. Ice block was sliced into sections of 5 *μ*m in thickness. Harris hematoxylin solution and eosin solution were used for staining. Neutral gum seal was observed under a light microscope.

### 2.9. Quantitative Real-Time PCR (qRT-PCR)

Total RNA was isolated from cells or tissues (100-200 mg) using TRIzol reagent (Invitrogen, USA), according to manufacturer's protocols. RNA quantity was measured using the NanoDrop TM 8000 spectrophotometer (Thermo Fisher Scientific, France). cDNA was synthesized using a reverse transcriptase kit (Roche, USA). Sequences of gene-specific Polymerase Chain Reaction (PCR) primers (Shanghai Generay Biotech Co. Ltd., China) used were as follows: IL-1*β*—5′-AAATGCCTCGTGCTGTCTGA-3′ (forward), 5′-AGGCCACAGGGATTTTGTCG-3′ (reverse); IL-6—5′-AAAGCCAGAGTCATTCAGAGCA-3′ (forward), 5′-GCATTGGAAGTTGGGGTAGGA-3′ (reverse); TNF-*α*—5′-TCTTCTCATTCCTGCTCGTGG-3′ (forward), 5′-TGATGAGAGGGAGCCCATTTG-3′ (reverse).

QRT-PCR was performed in 20 *μ*l volumes with SYBR Green PCR Master Mix (Roche, USA) at 95°C for 10 min and 40 cycles at 95°C for 15 s, 60°C for 30 s, and 72°C for 30 s, using Light Cycler 480 (Roche, USA). IL-1*β*, IL-6, and TNF-*α* levels were quantified with the 2-*ΔΔ*Ct; GAPDH mRNA was measured as an internal control.

### 2.10. Western Blot

Cells or tissue homogenates were extracted from the Petri dish and rat ventricles for immunoblotting analysis. The protein content was measured with bicinchoninic acid (BCA) Protein Assay Kit (Bio-Rad, Mississauga, ON, Canada). Each amount of protein (60 *μ*g) was resolved in 10% SDS-PAGE gel. The lysates were resolved by electrophoresis (70 V for 30 min and 100 V for 1.5 h) and transferred onto nitrocellulose membranes; then 5% nonfat milk was used to block for 2 h at room temperature. The membranes were incubated with primary antibodies at 4°C for overnight, washed three times with PBST (10 min/each), and incubated for 1 h with the secondary antibodies (1 : 4000) in the dark place. An equal input of protein samples used GAPDH as an internal control. Western blot bands were quantified using Odyssey v1.2 software by measuring the band intensity (area × OD) for each group. The final results are expressed as fold changes by normalizing the data to the control values.

### 2.11. Data Statistical Analyses

Data are expressed as the mean ± SEM. The statistical analysis of the results was performed with GraphPad Prism version 5.0 (GraphPad Software Inc., San Diego, CA, USA). Student's *t*-test was used for a two-group comparison. One-way ANOVA followed by Dunnett's *t*-test was used for a multiple-group comparison. *P* < 0.05 was considered statistically significant.

## 3. Results

### 3.1. AE Reduces Myocardial Inflammatory Injury in HFD

**Figure 1:**
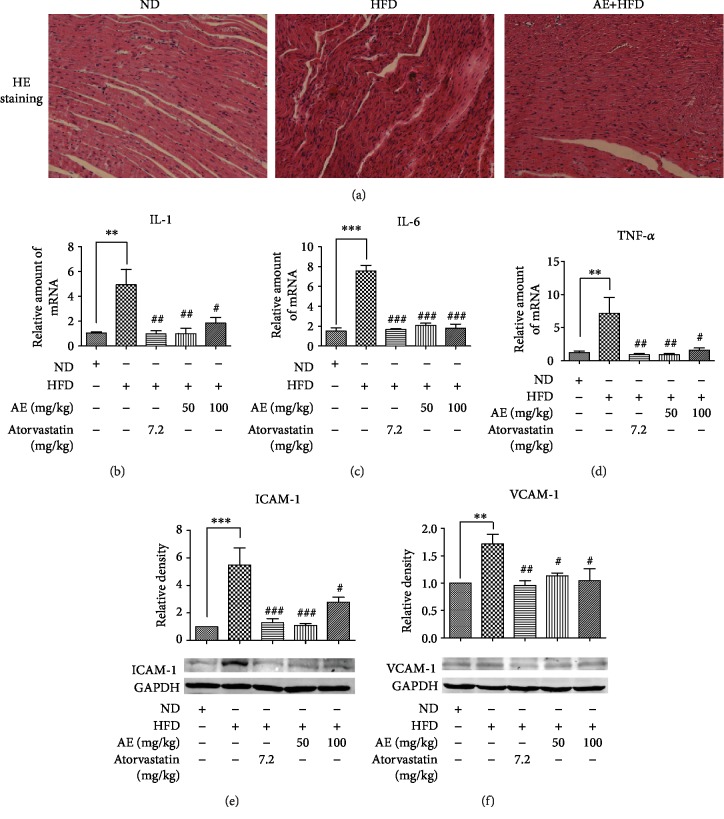
AE reduces myocardial inflammatory injury in HFD experimental model; HFD also increased the mRNA levels of IL-1*β*, IL-6, and TNF-*α*. Staining as described in the Material and Methods section. The frozen heart tissues were sectioned at 5 *μ*m for HE staining (*n* = 4). All images were obtained by an optical microscope with 200x amplification as shown in (a). The representative data of qRT-PCR analysis of RNA extracted from the heart are shown in (b)–(d). All the samples were normalized with GAPDH (*n* = 6). (e, f) The heart tissues in the control group, HFD group, HFD+atorvastatin group (positive control group), and HFD+AE group (the treatment group) were collected and homogenized to detect ICAM-1 and VCAM-1 level by western blot analysis as shown in (e) and (f). GAPDH was used as a loading control. The column figures show the normalized optical density of each protein (*n* = 5; ^∗∗^*P* < 0.01 and ^∗∗∗^*P* < 0.001 compared to the ND group; ^#^*P* < 0.05, ^##^*P* < 0.01, and ^###^*P* < 0.001 compared to the HFD group).

To assess the effect of AE on heart inflammation induced by HFD, we gathered the rat hearts at 10 weeks of feeding as described in the Material and Methods section. Firstly, we analyzed the impact of HFD on serum lipids. Compared with those in the ND group, the LDL, TG and TC circulatory levels were significantly higher in the HFD group (Supplementary [Supplementary-material supplementary-material-1]). Interestingly, 6-week treatment of AE reversed the hyperlipidemia and AE reduced LDL, TG, and TC circulatory levels induced by HFD, consistent with our previous study [17]. Increased inflammatory infiltration is one of the hallmarks of the progression of inflammation; hematoxylin-eosin staining validated our guess. The histological analysis of tissue in heart sections from Wistar rats showed an increased inflammatory infiltration in HFD compared with the ND group, while the inflammatory infiltration was reversed in the AE treatment group and ATO group (Figure 1(a)). A number of studies have illustrated that interleukin and tumor necrosis factor were the indicators of inflammatory response activation [18, 19]; we investigated the expression of these mRNAs by qRT-PCR. The data showed that HFD significantly increased the mRNA expression of IL-1*β* nearly fourfold compared with the ND group; treatment with AE and atorvastatin (ATO) significantly inhibited HFD-induced heart inflammatory cytokine expression. ATO is used as a positive control drug which has a great effect on lowering lipid levels. In addition, our data revealed that mRNA expressions of IL-1*β* in AE and ATO groups were not statistically significant which suggested that AE has similar efficacy to ATO in curing heart inflammation induced by HFD (Figure 1(b)). Surprisingly, there was no significant difference in IL-1*β* mRNA expression between the AE 50 mg/kg group and AE 100 mg/kg group. The expressions of TNF-*α* and IL-6 in HFD-fed rats exhibited the same trend; treatment with AE and ATO induced significantly suppressed mRNA expressions of TNF-*α* and IL-6 in HFD (Figures 1(c) and 1(d)). A similar effect of AE on the suppression of the HFD-induced protein expression of ICAM-1 and VCAM-1 vascular adhesion factor was observed in western blot, which are the major vascular adhesion factors and play an important role in inflammation [20]. To further confirm the effect of AE on inflammation induced by HFD, the expression of vascular adhesion factor was further detected. As is shown in Figures 1(e)and 1(f) , the HFD group had a higher expression of ICAM-1 and VCAM-1, with 6.5-fold upregulation of ICAM-1 and 1.8 upregulation of VCAM-1, respectively, indicating the activation of local inflammatory processes. These changes were remarkably blocked by AE administration. The data revealed that both AE and ATO could reduce the expression of ICAM-1 and VCAM-1 induced by HFD. However, AE resulted in significantly declined expression of vascular adhesion factor.

### 3.2. AE Inhibits PA-Induced Inflammatory Cytokine Production in H9C2 Cells

**Figure 2:**
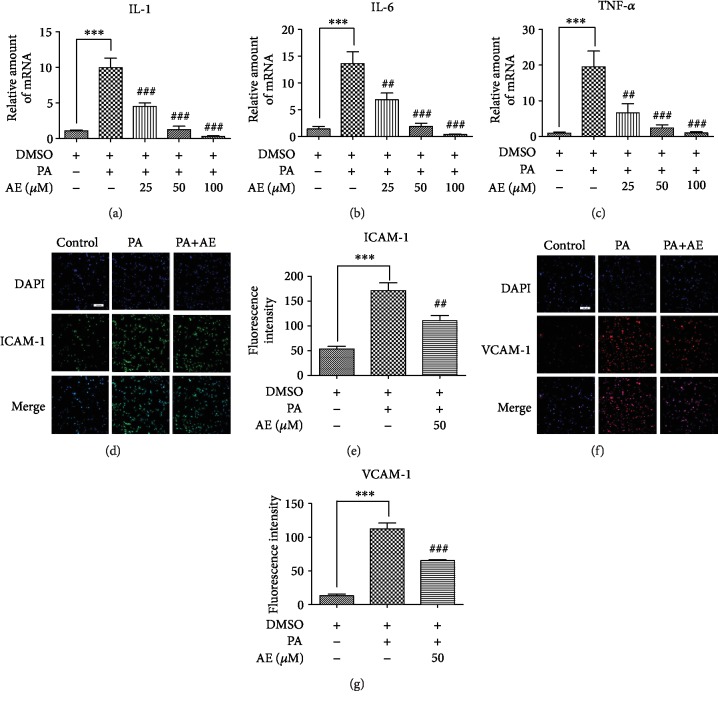
AE inhibits PA-induced inflammatory cytokine production in H9C2 cells. (a–c) H9C2 cells pretreated with different concentrations of AE for 1 h and incubated with 500 *μ*M PA for 14 h. The total RNAs were extracted from cells for real-time qPCR analysis as shown in (a)–(c). All the genes were normalized with GAPDH as a control (*n* = 6). (d, f) The cells were harvested and processed to immunofluorescence staining of vascular adhesion factor (ICAM-1 and VCAM-1) as described in the Material and Methods section. (e, g) Representative group fluorescence intensity was shown. Transverse line = 100 *μ*m (*n* = 4; ^∗∗∗^*P* < 0.001 compared to the control group; ^##^*P* < 0.01 and ^###^*P* < 0.001 compared to the PA group).

Previous studies have shown that palmitic acid (PA) induces an inflammatory phenotype in H9C2 cells. This inflammatory activity is characterized by increased production of proinflammatory cytokines and oxidants [21, 22]. The HFD-associated myocardial inflammatory injury was likely attributed to both direct and indirect effects of PA and other SFAs on cardiac cells [22]. In vitro, we first tested whether different concentrations of AE had any effect on the survival rate of H9C2 cardiomyocytes. Supplementary [Supplementary-material supplementary-material-1] shows that even at high concentrations of AE (400 *μ*M), there was no effect on the survival rate of cells. Then we employed the PA-induced inflammation production in H9C2 cardiac cells to investigate whether AE exhibits anti-inflammatory effects in hyperlipidemia. PA at the concentration of 500 *μ*M was used to challenge H9C2 cells. As shown in Figure 2(a), the expression of IL-1*β* was remarkably upregulated in the HFD group, with 10-fold upregulation; the treatment with different concentrations of AE significantly decreased IL-1*β* production induced by PA; AE pretreatment abrogates this increase in a dose-dependent manner. Likewise, the mRNA expression levels of IL-6 and TNF-*α* increased remarkably with PA stimulation; pretreatment with AE significantly reversed these changes in IL-6 and TNF-*α* production (Figures 2(b) and 2(c)). To further verify our observed phenomenon in H9C2 cells, the same validation was carried out in vitro. Immunofluorescence staining showed vascular adhesion factor expression was significantly increased in H9C2 cell combination treatments of PA (Figures 2(d) and 2(f)); vascular adhesion factor changes manifested the increased mean fluorescence intensity levels of ICAM-1 and VCAM-1 (Figures 2(e) and 2(g)), but pretreatment of cells with AE effectively attenuated the PA-increased vascular adhesion factor of fluorescence intensity levels. These results clearly support the notion that AE plays a key role in mediating PA-induced inflammatory injury.

### 3.3. AE Enhanced H9C2 Cell Viability and Reduced Cell ROS Production after PA Challenge

**Figure 3:**
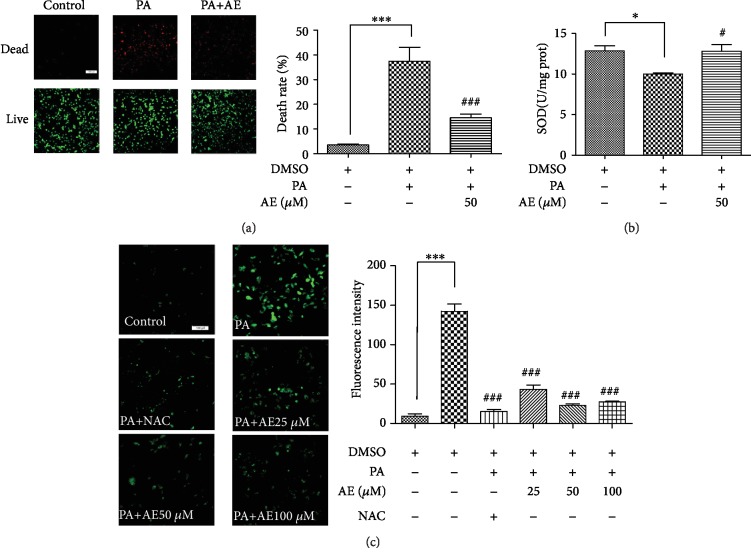
AE enhanced H9C2 cell viability and reduced ROS production after PA challenge. H9C2 cells pretreated with different concentrations of AE for 1 h and incubated with PA (500 *μ*M) for 14 h were used for calcein-AM/PI staining, DCFH-DA assay, and investigation of SOD expression. The images from fluorescence microscopy and data from the microplate reader are shown in (a)–(c). Data are expressed as the mean ± SD. Transverse line = 100 *μ*m (*n* = 4; ^∗^*P* < 0.05 and ^∗∗∗^*P* < 0.001 compared to the control group; ^#^*P* < 0.05 and ^###^*P* < 0.001 compared to the PA group).

Existing studies have found that HFD-induced lipid accumulation and oxidative stress promote inflammation [23, 24]; this prompted us to investigate the removing activity of AE on PA-induced reactive oxygen species (ROS) production in H9C2 cells. Therefore, we examined the effect of AE on the oxidative stress and the amount of live and dead rate of H9C2 cells challenged by PA. Our results show that PA was able to promote cell death and AE significantly improved cell survival under the PA stimulation (Figure 3(a)). We then examined whether PA-induced inflammation was associated with oxidative stress. Antioxidative index, for example, SOD, a natural scavenger of oxygen free radicals in living organisms was investigated. The PA-prevented SOD activity was clearly improved in H9C2 cells. However, the cellular SOD level was evidently increased in H9C2 with AE treatment after PA stimulation (Figure 3(b)). Inversely, as illustrated in Figure 3(c), PA treatment upregulated the generation of ROS in H9C2 cells; we observed that PA stimulated ROS to release into the culture media, whereas being abolished by treatment with different concentrations AE and N-acetyl-L-cysteine. Together, the findings above indicated that AE alleviated PA-induced oxidative stress and increased cell survival efficiency.

### 3.4. AE Administration Inhibits TLR4 Pathway Signaling in HFD-Induced Hyperlipidemia

**Figure 4:**
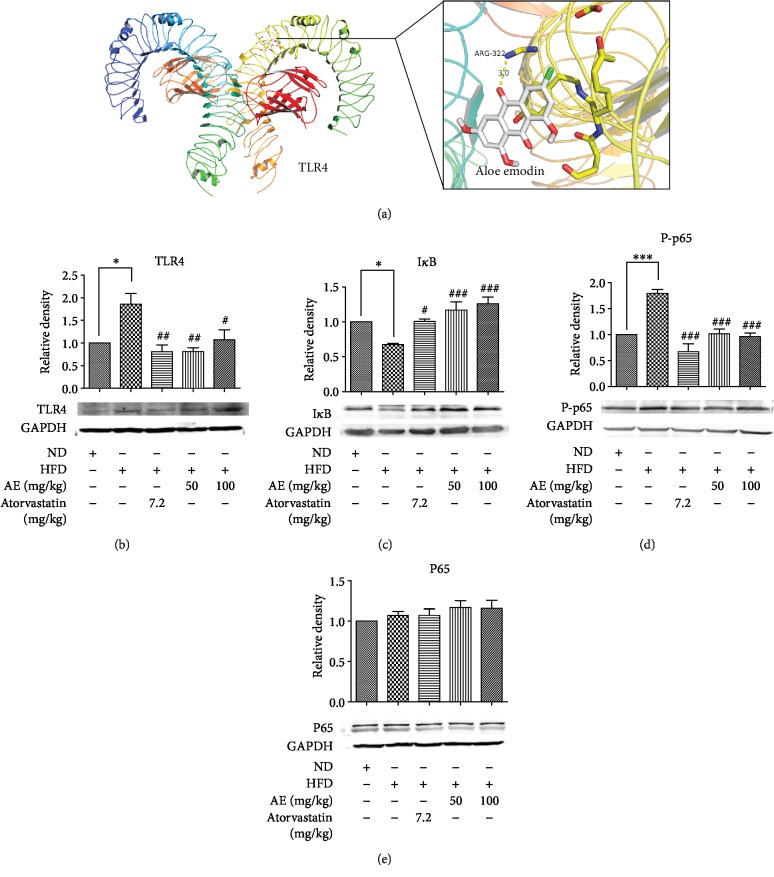
AE has an important repressor effect on the HFD-stimulated TLR4 pathway signaling. 3D schematic diagram of interaction between AE and TLR4 active site residues: the yellow dotted line is hydrogen bonding (a). Heart tissue homogenate was extracted for western blot assay. GAPDH was used as a loading control. The column figures have shown the normalized optical density of each protein as follows: (b) Toll-like receptor 4 (TLR4), (c) inhibitor of NF-*κ*B (I*κ*B), (d) nuclear factor-kappa B P65 (NF-*κ*B P65), and (e) p-nuclear factor- kappa B P65 (p-NF-*κ*B P-P65) (*n* = 5; ^∗^*P* < 0.0 and ^∗∗∗^*P* < 0.001 compared to the ND group; ^#^*P* < 0.05, ^##^*P* < 0.01, and ^###^*P* < 0.001 compared to the HFD group).

Recent studies showed an association between the increase in TLR4 levels and an increase in lipid levels, which suggests that TLR4 are important in hyperlipidemia [12]. Besides, hyperlipidemia could cause inflammation and fibrosis in H9C2 cells and heart tissue [25]. In order to further understand HFD-induced inflammation changes after AE treatment, the effect of AE on critical components of the TLR4 signaling pathway was examined. Firstly, we used BATMAN-TCM (https://bionet.ncpsb.org/batman-tcm/index.php/Home/Index/index) [26] to predict AE targets, and we found that TLR4 could be a potential target of AE. After that, AutoDock Vina [27] was used for docking; the 3D structure diagram of protein and ligands was generated by PyMol. The results showed that AE formed hydrogen bond interaction with the active microstore residue ARG-322 of TLR4 and the hydrogen bond length was 3 Å (Figure 4(a)). Furthermore, our present analysis indicates that HFD increases the expression of TLR4 (Figure 4(b)) and AE could reverse the protein expression of TLR4 induced by HFD. Then, critical downstream signaling targets of the TLR4 were evaluated with western blot analysis. An activated TLR4 recruits MyD88, thereby causing phosphorylation of the inhibitor of *κ*B (I*κ*B) [11]; we assayed for I*κ*B levels to determine whether AE alters nuclear factor-*κ*B (NF-*κ*B) activation in HFD. NF-*κ*B is a downstream target of the TLR4 pathway and regulates the expression of proinflammatory genes. Our data identified that the protein level of I*κ*B-*α* was decreased in the HFD group compared with the ND group; AE not only inhibited the expression of TLR4 but also inhibited HFD-induced I*κ*B phosphorylation (Figure 4(c)). Activated TLR4 can subsequently activate the downstream NF-*κ*B pathway and promote the synthesis and release of IL-1*β* and TNF-*α* [11]. To confirm the reliability of the results, we studied the phosphorylation of NF-*κ*B. As expected, HFD increased phosphorylation levels of NF-*κ*B p65; meanwhile, HFD stimulation of NF-*κ*B p65 phosphorylation was inhibited by AE and ATO (Figures 4(d) and 4(f)). Thus, the findings above indicated that AE does exert an anti-inflammatory effect through affecting the HFD-stimulated TLR4 signaling pathway.

### 3.5. AE Has an Important Repressor Effect on the PA-Induced TLR4 Pathway Signaling

**Figure 5:**
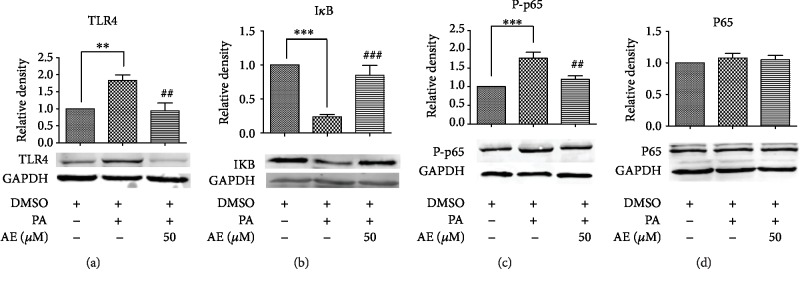
AE has an important repressor effect on the PA-induced TLR4 pathway signaling. The extracted total proteins were processed to western blot analysis with GAPDH as the loading control. The column figures demonstrated the normalized optical density of (a) Toll-like receptor 4 (TLR4), (b) inhibitor of NF-*κ*B (I*κ*B), (c) nuclear factor-kappa B P65 (NF-*κ*B P65), and (d) p-nuclear factor-kappa B P65 (p-NF-*κ*B P-P65) proteins (*n* = 5; ^∗∗^*P* < 0.01 and ^∗∗∗^*P* < 0.001 compared to the control group; ^##^*P* < 0.01 and ^###^*P* < 0.001 compared to the PA group).

Many studies have also shown that SFAs induce inflammatory phenotypes in H9C2 cells through TLR4 [28]. We also observe that the PA augment TLR4 expression, and the augmentation was mediated by the nuclear factor-*κ*B (NF-*κ*B) pathway [29]. To further verify the anti-inflammatory mechanism of AE, several key TLR4 signaling pathway proteins were examined. The results indicate high expression of TLR4 in PA challenges and AE treatment decreased TLR4 protein expression (Figure 5(a)). In addition, consistent with our findings in vivo, our results show that PA could degrade I*κ*B-*α* protein expression (Figure 5(b)) and increase phosphorylation of NF-*κ*B p65 (Figures 5(c) and 5(d)); AE prevented PA-induced I*κ*B-*α* degradation and phosphorylation of the NF-*κ*B p65. The findings indicated that PA activates TLR4 signaling pathways, resulting in the activation of NF-*κ*B signaling for the regulation of proinflammatory molecules; pretreatment with AE at 50 *μ*M significantly reversed these changes induced by PA.

## 4. Discussion

At present, hyperlipidemia is one of today's most obvious public-health problems due to its high prevalence and its association with metabolic syndromes, such as chronic inflammation, insulin resistance, and NAFLD [6]. Chronic inflammation in association with insulin resistance is a key characteristic of hyperlipidemia. Excessive intake of HFD may result in hyperlipidemia, leading to blood lipid metabolism disorder and chronic inflammation, ultimately contributing to the progression of arterial vascular stenosis [30, 31]. Increased morbidity of chronic inflammation caused by HFD in children with type 2 diabetes mellitus is considered to be the result of this obesity epidemic [32, 33]. It is urgent to find the measures for effective treatment.

From ancient times to modern times, many traditional drugs from plants have played an important role in the treatment of human diseases. For decades, synthetic chemicals have been effective in the treatment of most diseases; in recent years, some bioactive compounds such as isoflavones and diosgenin, which are usually obtained from terrestrial plants, have been proved to reduce the risk of cardiovascular diseases and also help to protect the heart [34]. The structure-function relationship of phytochemicals could play a leading role in future cardiovascular drug design. AE is an anthraquinone monomer with extensive pharmacological effects, it has an R2 hydroxymethyl, and the structural characteristics of the anthraquinone suggest that acidic substitution with a phenolic or carboxylic group at R1 or R2 position, or polar, hydrophilic substitution such as hydroxymethyl group at R2 position may contribute to the anti-inflammatory potency [35]. Accumulating evidence has demonstrated that the desirable preventive or therapeutic effects of AE are associated with its anti-inflammatory properties. In light of our knowledge, our study is the first to explore the role of AE in HFD-induced cardiac inflammation.

In the present study, we established a hyperlipidemia model; the hyperlipidemia model was induced by HFD treatment in Wistar rats and PA treatment in H9C2 cells as described previously. In this model, HFD and PA have a role in the generation of inflammatory factors associated with hyperlipidemia. A prominent feature of HFD-triggered hyperlipidemia is the accumulation of lipid, as well as promoting low-grade chronic inflammation associated with increased levels of mediators such as IL-1*β*, IL-6, and TNF-*α* [36]. IL-1*β* is the most studied member of the IL-1 family due to its role in mediating autoinflammatory diseases [37]; it has been reported that hyperlipidemia could result in the accumulation of apoptotic bodies, followed by a robust IL-1-dependent increase in serum inflammatory cytokines [38]. IL-6 and TNF-*α* are proinflammatory cytokines with extensive effects; they come from macrophages, lipocytes, or endotheliocytes. The physically normal IL-6 and TNF-*α* levels in the blood of healthy people have the effects of anti-infection and immune function enhancement, but too much of them can damage the tissue [39]. Our experimental study found that, under the HFD and PA stimulation, the expression levels of IL-1*β*, IL-6, and TNF-*α* showed a significant upward trend compared with the control group. However, AE plays an important role in treating heart inflammation caused by hyperlipidemia; AE-treated groups displayed a significant reduction in the proinflammatory profile found in Wistar rats and H9C2 cells.

Intercellular cell adhesion molecule 1 (ICAM-1) and vascular cell adhesion molecule 1 (VCAM-1) are two important members of the immunoglobulin gene superfamily but play different roles in the adhesion of leukocytes to the vascular endothelium. Many clinical investigators have measured the levels of soluble ICAM-1 and VCAM-1 as the potential biomarkers for endothelial dysfunction and early atherosclerosis [40]. ICAM-1 is a glycoprotein, which is generally expressed on the cell membranes of vascular endothelium and other cells. It is regulated by endotoxin and some cytokines including TNF-*α*, IL-1*β*, and IFN-*γ* in vitro and in vivo; ICAM-1 plays an important role in promoting adhesion at the site of inflammation, controlling tumor progression and metastasis, and regulating immune responses in the body [41]. It creates conditions for white blood cells to bind tightly to endothelial cells and mediate their migration into the endothelium to participate in various physiological and pathological processes [42]. An early feature of inflammation is the release of cytokines leading to the increased expression of endothelial activation markers such as VCAM-1 [43]. These membrane proteins are necessary for anchoring leukocytes to the vessel wall and are well-established markers in inflammatory conditions such as hyperlipidemia. We observed a significant augmentation of ICAM-1 and VCAM-1 when rats were treated with HFD and H9C2 cells stimulated with PA. AE significantly alleviated the expression of ICAM-1 and VCAM-1 induced by HFD/PA, in both in vitro and in vivo.

Oxidative stress plays a critical role in the pathogenesis of various diseases [44]; many chronic diseases are characterized by excessive oxidative stress and inflammation [45, 46]. Increased oxidative stress is also the basis of pathophysiology of hypertension and atherosclerosis by directly affecting vascular wall cells [47]. Previous studies have established that elevations in glucose and free fatty acids often associated with obesity and hyperlipidemia which could promote the generation of ROS in adipocytes. Increased oxidative stress in accumulated fat is an important pathogenic mechanism of obesity-associated inflammation [48]. In addition, it is well known that oxidative stress and inflammation in cells are strongly associated with apoptosis [49]. In this study, immunofluorescence and kit experiments showed that AE could significantly reduce the production of ROS induced by PA and increase the expression of SOD. Our findings support that AE treatment decreases cardiomyocyte apoptosis; it is reasonable to speculate that AE alleviated apoptosis by alleviating the oxidative stress and inhibiting cell death.

Potential molecular links between FFAs and metabolic diseases are the Toll-like receptors; FFAs trigger inflammatory responses via TLR4 promoting inflammatory gene expression by triggering NF-*κ*B activation [50, 51]. NF-*κ*B is a dimeric transcription factor located in the cytoplasm of nonstimulated cells because of interactions with inhibitory proteins such as I*κ*Bs; inflammatory stimuli induce the phosphorylation of the I*κ*B kinase (IKK) complex to I*κ*B*α*, which leads to ubiquitination and proteasome-mediated degradation of I*κ*B*α* [52]. Following free NF-*κ*B phosphorylation translocating to the nucleus, the NF-*κ*B heterodimer activates the transcription of target genes, including multiple inflammatory genes, such as IL-1*β*, IL-6, TNF-*α*, and cell adhesion molecules [53]. AE exerts its anti-inflammatory effects via several mechanisms [35]; our studies showed that HFD increases TLR4 expression and NF-*κ*B activity in the heart tissues of mice and H9C2 cells, while decreasing I*κ*B expression. AE significantly inhibited TLR4 and NF-*κ*B activation induced by PA/HFD thus alleviating the expression of inflammatory cytokines like TNF-*α*, IL-6, and IL-1*β*, both in vitro and in vivo. Besides, with the molecular docking analysis, we found that AE could affect the identification of TLR4 lipids by occupying the active sites of TLR4 which supports the fact that AE inhibits HFD-induced cardiac inflammation via inactivation of TLR4.

In conclusion, the findings of the present study in both in vitro and in vivo reinforce the defensive role of AE against oxidative stress, inflammation, and cell death. The beneficial actions of AE are closely associated with its ability to decrease TLR4 and inhibiting NF-*κ*B signaling pathways. These results provide a deeper understanding of the regulatory role of TLR4 and NF-*κ*B for HFD-induced cardiac injury; it suggests that they may be important therapeutic targets for obesity-related diseases.

## Data Availability

The data used to support the findings of this study are available from the corresponding author upon request.

## Abbreviations

ATO: Atorvastatin
AE: Aloe emodin
BCA: Bicinchoninic acid
BSA: Bovine serum albumin
DMEM: Dulbecco's modified Eagle medium
DAPI: 4′,6-Diamidino-2-phenylindole
HFD: High-fat diet
HDL: High-density lipoprotein
ICAM-1: Intercellular cell adhesion molecule 1
IL-1*β*: Interleukin-1
IL-6: Interleukin-6
I*κ*B: An inhibitor of NF-*κ*B
LDL: Low-density lipoprotein
ND: Normal diet
NF-*κ*B p65: Nuclear factor-kappa B P65
p-NF-*κ*B P65: p-nuclear factor-kappa B P65
PA: Palmitic acid
PBS: Phosphate-Buffered Saline
PBST: Phosphate-Buffered Saline Tween-20
PCR: Polymerase Chain Reaction
SDS-PAGE: Sodium dodecyl sulphate-polyacrylamide gel electrophoresis
SFAs: Saturated fatty acids
ROS: Reactive oxygen species
SOD: Superoxide Dismutase
TLR4: Toll-like receptor 4
TLR2: Toll-like receptor 2
TC: Total cholesterol
TG: Triglyceride
TNF-*α*: Tumor necrosis factor-*α*
VCAM-1: Vascular cell adhesion molecule 1.

## Supplementary Materials

Supplementary Materials Supplementary Figure 1: HFD could elevate blood lipids in Wistar rats. Mice were grouped and treated with normal diet and high-fat diet for 4 weeks. Then the serums were collected and used for the measurement of HDL-C (A), TG (B), TC (C), and LDL-C (D) levels. These data are presented as the means ± SEM. ^∗^Significant difference at *P* < 0.05, ^∗∗^significant difference at *P* < 0.01, and ^∗∗∗^significant difference at *P* < 0.001 vs. the ND group. Supplementary Figure 2: effects of different AE concentrations on H9C2 cell viability by CCK-8 assay. The cells were treated as designated in 96-well plates. Next, the cells in each well were incubated with different concentrations of AE for 14 h. Finally, 10 *μ*l of cck-8 is added to each hole. The culture plates were shook for 1 min, and the absorbance was at 450 nm.
