# Supplementary material for: Aloe Emodin Reduces Cardiac Inflammation Induced by a High-Fat Diet through the TLR4 Signaling Pathway
Source: Mediators Inflamm. 2020 Feb 5;2020:6318520. doi: 10.1155/2020/6318520 (PMC7025072; doi:10.1155/2020/6318520)
Supplement: Supplementary Materials — Supplementary Figure 1: HFD could elevate blood lipids in Wistar rats. Mice were grouped and treated with normal diet and high-fat diet for 4 weeks. Then the serums were collected and used for the measurement of HDL-C (A), TG (B), TC (C), and LDL-C (D) levels. These data are presented as the means ± SEM. ∗Significant difference at P < 0.05, ∗∗significant difference at P < 0.01, and ∗∗∗significant difference at P < 0.001 vs. the ND group. Supplementary Figure 2: effects of different AE concentrations on H9C2 cell viability by CCK-8 assay. The cells were treated as designated in 96-well plates. Next, the cells in each well were incubated with different concentrations of AE for 14 h. Finally, 10 μl of cck-8 is added to each hole. The culture plates were shook for 1 min, and the absorbance was at 450 nm. [file 6318520.f1.docx]

Supplemental data


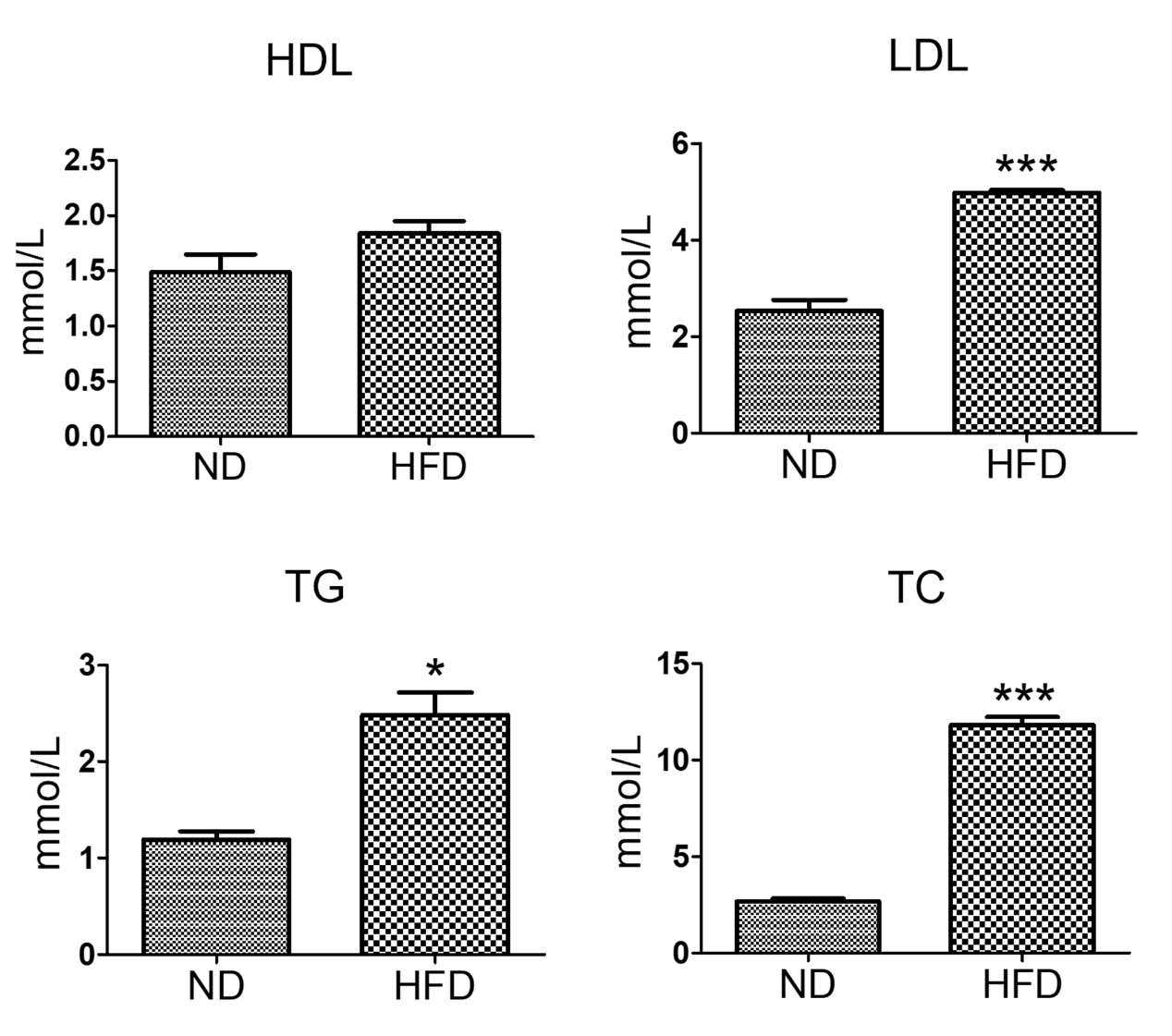


**Supplemental Figure 1. HFD could elevates blood lipids in Wistar rats.** Mice were grouped and treated with normal diet and high fat diet for 4 weeks.  Then the serums were [collection](javascript:;) used for the measurement of HDL-C, LDL-C, TG and TC levels. These data are presented as the means ±SEM. (*) Significant difference at *p* < 0.05, (**) significance difference at *p* < 0.01 and (***) significance difference at *p* < 0.001 VS the ND group.


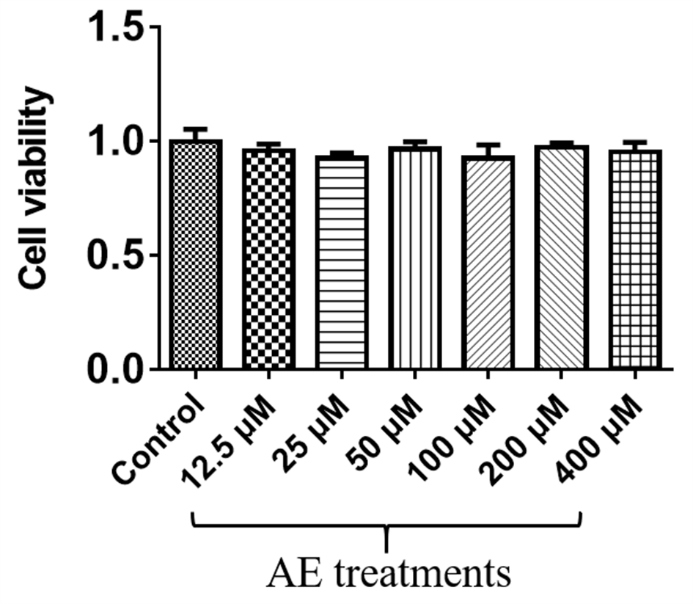


**Supplementary figure 2: Effects of different AE concentrations on H9C2 cell viability by CCK-8 assay.** The cells were treated as designated in 96-well plates. Next, the cells in each well were incubated with different concentrations of AE for 14 h. Finally, 10μl of cck-8 is added to each hole. The culture plates were shook for 1 min, and the absorbance at 450 nm.
